# Hallachrome, a marine anthraquinone, impairs cell proliferation by acting as a functional mitochondrial uncoupler in A375 melanoma cells

**DOI:** 10.3389/fphar.2026.1844144

**Published:** 2026-07-27

**Authors:** Francesco Demetrio Lofaro, Anita Ferri, Alessia Mazzilli, Susanna Bonacorsi, Roberto Simonini, Federica Boraldi

**Affiliations:** Department of Life Sciences, University of Modena and Reggio Emilia, Modena, Italy

**Keywords:** anthraquinones, hallachrome (HC), marine drug, melanoma, mitochondria, natural product, proteomic

## Abstract

**Background:**

Melanoma remains one of the most lethal cancers due to its high metastatic capacity and resistance to current therapies, emphasizing the need for novel antineoplastic agents, including bioactive molecules from marine organisms, as potential sources of anticancer compound.

**Aims:**

This study aimed to investigate, for the first time, the anticancer potential of Hallachrome (HC), an anthraquinone derived from the marine polychaete *Halla parthenopeia*, in A375 melanoma cells.

**Methods:**

A375 melanoma cells were treated with HC *in vitro* to evaluate its impact on cell proliferation, migration, and morphology. Proteomic analysis was carried out to identify differentially expressed proteins in order to elucidate the biological processes modulated by the treatment. Mitochondrial morphology and function were analyzed by confocal microscopy and oxygen consumption rate, respectively, while mitochondrial membrane potential, intracellular reactive oxygen species (ROS) levels and GSH/GSSG ratio were evaluated by fluorescence-based assays.

**Results:**

HC treatment significantly reduced melanoma cell proliferation and migration *in vitro*, accompanied by morphological alteration and apoptosis death. Proteomic profiling revealed differential expression of several proteins involved, for example, in membrane and mitochondria organization. Moreover, HC treatment induced severe mitochondrial dysfunction, characterized by ATP depletion, mitochondrial membrane depolarization, fragmentation of the mitochondrial network, altered intracellular ROS level and GSH/GSSG ratio.

**Discussion:**

These findings suggest that HC inhibits melanoma cell survival by disrupting mitochondrial homeostasis and inducing energetic stress. Given the pivotal role of mitochondria in cancer progression, the ability of HC to interfere with mitochondrial functions supports its potential as a promising anticancer agent.

## 1 Introduction

Melanoma is one of the most aggressive cancers due to its high metastatic potential and mortality rate (Davis et al., 2019). Despite significant progress in treatment, many patients develop drug resistances or fail to respond to therapies, underscoring the need to discover new anticancer agents (Davis et al., 2019). Natural products derived from both terrestrial and marine organisms represent a fundamental source of bioactive compounds for drug development (Chopra and Dhingra, 2021). In particular, marine organisms are an abundant and underexplored reservoir of novel compounds with potential applications for human health. Since 1980s, hundreds of new marine-derived molecules have been discovered each year (Greco and Cinquegrani, 2016), many of which exhibit antibacterial, antiviral, anti-inflammatory and anticancer activity (Molinski et al., 2009; Mayer et al., 2022; Pecoraro et al., 2023). To date, approximately fifteen marine-derived compounds (MDCs) have been approved for clinical use (e.g., Carragelose®, Retrovir®, Prialt®, and Yondelis®) (Arrieta et al., 2010). Interestingly, MDCs tend to exhibit greater cytotoxicity compared to their terrestrial counterparts, making them excellent candidates for cancer therapies due to their affordability and availability (Proksch et al., 2003; Chaudhry et al., 2022). Currently, 9 MDCs have been approved as anticancer drugs, and 19 are being evaluated in clinical trials (Zuo and Kwok, 2021), with only two tested for melanoma.

Anthraquinones represent a class of molecules that can be isolated from both plant and marine organisms, and they have been shown to possess remarkable biological activities (Choi et al., 2013; Chien et al., 2015; Diaz-Muñoz et al., 2018; Li and Jiang, 2018).

Some plant- and lichen-derived anthraquinones (e.g., Aloin; Purpurin; Parietin, Emodin) could potentially be employed in cancer treatment (Solhaug and Gauslaa, 1996; Chen et al., 2013; Kumar et al., 2017; Tian et al., 2020; Wang W. et al., 2021; Nowak-Perlak et al., 2023), because they inhibit cancer cell proliferation, migration and invasion (Semwal et al., 2021), induce cell death (Tian et al., 2020) and some of them possess antioxidant and anti-inflammatory activities (Siddamurthi et al., 2020). However, their extraction process is often challenging, their content in plants is often low, and they can also induce toxic side-effects. Marine-derived anthraquinonoid compounds have also demonstrated a variety of biological activities (Greco et al., 2021). Interestingly, Hallachrome (HC; 7-hydroxy-8-methoxy-6-methyl1,2-anthraquinone, C_16_H_12_O_4_) (Prota et al., 1971; Cameron et al., 1999), an anthraquinone with a unique structure isolated from the defensive mucus produced by specific epidermal cell of polychaete *Halla parthenopeia* (Ferri et al., 2024a), has demonstrated biological effects against pathogenic microbes (*Candida albicans*, *Staphilococcus aureus*) and small invertebrates used in ecotoxicity tests (Simonini et al., 2019; Ferri et al., 2024c). Moreover, this substance can be easily collected and purified without the need to sacrifice animals, which can be raised in a controlled aquarium system (Ferri et al., 2024b). Unlike other plant anthraquinones, its potential effects on cancer cells have not yet been explored.

Given the growing interest in marine-derived anthraquinones and the lack of data on the potential anticancer properties of HC, aim of this study was to investigate the effect of HC on highly proliferative A375 human melanoma cells by evaluating i) cell proliferation and morphology; ii) antimigration ability; iii) proteome profile; iv) redox state and v) mitochondrial function.

## 2 Materials and methods

### 2.1 Worms’ maintenance, mucus collection and hallachrome extraction

*Halla parthenopeia* is a large marine annelid (up to 1 m in length) that burrows into the sandy bottoms of the Mediterranean. It is marketed live as bait and can be kept in the laboratory for years. When disturbed, it emits large quantities of purple mucus due to the presence of hallachrome. The latter can be obtained as pure crystals from the mucus by extraction with dichloromethane and subsequent chromatographic purification with a 4:1 toluene-ethyl acetate mixture. Details on the worm’s biology, collection and laboratory maintenance methods, and extraction, purification and analysis procedures have already been described (Simonini et al., 2019; Ferri et al., 2024a; 2024c; 2024b). Due to poor solubility of HC in aqueous solutions, the compound was dissolved in dimethyl sulfoxide (DMSO), at a 0.1% final concentration.

### 2.2 Cell lines

A375, SK-MEL-28 melanoma cell line and primary human dermal fibroblasts (HDFs) were purchased from Cytion (A375: 300,110, RRID: CVCL_0132; SK-MEL-28: 300,337, RRID: CVCL_0526; HDFs: 300606, Cytion, Heidelberg). The cells were cultured at 37 °C and 5% CO_2_ using high glucose Dulbecco’s Modified Eagle Medium (DMEM; Gibco, Grand Island, NY, United States) supplemented with 10% fetal bovine serum (Gibco), 1% non-essential amino acids (Gibco), 1% penicillin/streptomycin (Gibco).

### 2.3 IC_50_ and proliferation assay

A375 melanoma cells (5 × 10^4^) were seeded and grown in 12 wells in DMEM for 24 h. To evaluate the IC_50_, the culture medium was removed, and the cells were cultured for additional 24 h with: i) complete DMEM; ii) complete DMEM containing 0.1% DMSO; iii) complete DMEM containing HC at various concentrations (i.e., 1, 2, 4, 8, 10, 20 and 50 μM) all containing 0.1% DMSO (as in all experiments described below). Then, 24 h after treatment, cells were manually counted using a Neubauer chamber and observed with a phase-contrast optical microscope.

The concentration of HC able to inhibit cell viability by 50% (i.e., IC50) was calculated using the least square fit of four parameter-sigmoidal curves executed using GraphPad Prism (v. 9.5.0, San Diego, CA, USA). The concentration of HC able to inhibit A375 cell viability by 50% (i.e., IC_50_) was used in further experiments.

To assess proliferation, 3 cell lines were manually counted at specific time points (i.e., 4, 6, and 24 h for melanoma cells and an additional 48 h for HDFs) in different experimental conditions: (i) complete DMEM; (ii) complete DMEM containing 0.1% DMSO; and (iii) complete DMEM supplemented with 8.4 μM HC (i.e., IC_50_).

### 2.4 Morphological analysis

A375 human cells (1.5 × 10^5^) were seeded in 60 mm plates and grown in standard conditions (see paragraph cell culture) for 24 h at 37 °C with 5% CO_2_. Subsequently, the cells were cultured for additional 4 h with: i) complete DMEM; ii) complete DMEM containing 0.1% DMSO; iii) complete DMEM containing HC. After treatment, cells were washed two times with phosphate-buffered saline (DPBS; ThermoFisher Scientific, Waltham, MS, United States) and fixed with cold methanol for 10 min. Cells were stained with 0.5% of crystal violet in 20% of methanol aqueous solution for 10 min at room temperature. Images were acquired with bright-field light microscope (Zeiss AxioPhot). For each experimental condition 350 cells were analyzed using ImageJ software (v.1.54f). The following shape descriptors were considered: 1) surface area; 2) external perimeter; 3) circularity [4π·(area)/(perimeter^2^)]; 4) the ratio between areas of nucleus and cytoplasm; 5) aspect ratio [(major axis)/(minor axis) of each cell]; 6) roundness [4·(area)/π·(major axis^2^)]; 7) solidity; 8) nuclear area factor (NAF) (Daniel and DeCoster, 2004; Zanini et al., 2022).

### 2.5 Wound healing assay and transwell assay

A375 human melanoma cells (4 × 10^5^) were seeded in 6 well plate 24 hours before the assay. Wound was made with a sterile plastic tip and subsequently cells were incubated with complete DMEM or with 0.1% DMSO or with HC for additional 24 h. The melanoma cells were photographed under EVOS M5000 microscope (ThermoFisher Scientific) at the beginning (time point T0) and after 4, 8 and 24 h. Wound healing was evaluated at each time point by calculating the area of wound width using the ImageJ software (v.1.54f).

The migration assay was performed according Guy J-B and collaborators (Guy et al., 2017). Briefly, 2,5 × 10^5^ cells were seeded in 60 cm dishes and incubated for 24 h at 37 °C and 5% CO_2_. Cells were treated with DMSO and HC as described in the previous experiments. After 4 h of treatment, cells were detached using the cell scraper and 2,5 × 10^5^ cells were seeded in the upper chamber of Transwell chambers equipped with 5-μm pore-size polycarbonate membranes, as already described (Pijuan et al., 2019). To stimulate cell migration, the lower chambers were filled with complete culture medium. Cells were then incubated for 16 h at 37 °C with 5% CO_2_. Following incubation, cells were fixed with cold methanol and stained with crystal violet for 15 min at room temperature. Cells that had not migrated and remained on the upper surface of the membrane were gently removed with a cotton swab. Migrated cells on the lower surface of the membrane were observed using a bright-field light microscope (Zeiss AxioPhot) and quantified by counting cells in multiple representative fields using ImageJ software.

### 2.6 Apoptosis assay

A375 human melanoma cells (1 × 10^4^) were seeded into white 96-well plates and cultured for 24 h at 37 °C with 5% CO_2_. Then, cells were treated with DMEM, DMSO or 8.4 μM HC. Apoptosis was evaluated after 4 and 24 h of treatment using the Promega apoptosis assay kit (JA1011), according to the manufacturer’s protocol. Luminescence signal was measured using GloMax Explorer microplate reader (Promega Madison, WI, USA).

### 2.7 Protein extraction

A375 human melanoma cells (4 × 10^5^) were seeded in six well plate 24 h before the assay. After 4 h of treatment with complete DMEM containing 0.1% DMSO or HC, the cellular monolayer was washed two times with DPBS, detached using 0.5% trypsin-EDTA and centrifuged at 1,000 g for 10 min. The cellular pellets were stored at −80 °C until the use. Frozen cells were lysed in modified RIPA buffer (1% NP-40; 150 mM NaCl; 1% SDS and protease inhibitor cocktail), homogenized using a G19 needle and then incubated for 30 min in ice, as previously described (Zanini et al., 2022). Cellular debris were separated and discarded through centrifugation at 14,000 rpm for 10 min at 4 °C. The resulting supernatant was then transferred to a new, clean tube. The protein concentration was measured using the Bradford method (Bradford, 1976). Then, 100 µg of proteins from each sample were subjected to the filter aided sample preparation method (FASP) (Wiśniewski, 2018). Trypsin (v5280, Promega, Madison, WI, United States) was then used to digest proteins into peptides (1:50 ratio of trypsin to protein (wt/wt)) and the tryptic digests were suspended in water/acetonitrile/formic acid (95:3:2) solution and analyzed in mass spectrometry.

### 2.8 Mass spectrometry analysis

Peptides were injected into a Nano UHPLC Ultimate 3,000 system (Thermo Fisher Scientific) connected to Exploris™ 480 Hybrid Quadrupole-Orbitrap™ Mass Spectrometer (Thermo Fisher Scientific). Separation of the digests was achieved by employing a C18 EASY-Spray HPLC column (75 μm × 500 mm, 2 μm, ES903 Thermo Fisher Scientific) with a linear gradient ranging from 4% to 90% acetonitrile at a flow rate of 300 nL per minute (nL/min) over a duration of 150 min. During acquisition, the instrument operated in full MS/data-dependent acquisition mode. The Orbitrap mass analyzer was utilized at a resolution of 120,000 to capture full MS data in the mass-to-charge ratio (m/z) range of 400–1,500. For MS/MS analysis, fragments were measured at an Orbitrap resolution of 15,000. Twenty of the most intense ions were isolated for MS/MS analysis. The resulting raw data were processed using Proteome Discoverer software (version 3.0 SP1, Thermo Fisher Scientific). The database used for protein identification contained the human proteome retrieved from Uniprot (accessed in November 2022, containing 20,327 sequences). Several parameters were selected for protein identification, including: (i) the presence of at least one unique peptide; (ii) the static modification of carbamidomethyl on cysteines (+57.021 Da), the dynamic modifications of oxidation on methionine (+15.995 Da) and deamidation on asparagine and glutamine (+0.984 Da); (iii) a precursor mass tolerance of 10 parts per million (ppm) and a fragment mass tolerance of 0.02 Da; (iv) a maximum of one missed trypsin cleavage site; (v) a minimum peptide length of 7 amino acids. The database search analysis was performed using Sequest HT, and an artificial intelligence node called Chimerys was utilized to enhance identification. Label free quantification based on precursor ion intensity was performed using nodes implemented in Proteome Discoverer software: i) Minora Feature Detector node to detect peak; ii) Feature Mapper node for chromatographic alignment of precursors; iii) Precursor Ions Quantifiers with the following settings: Unique and Razor peptides, total peptide amount as normalization method, pairwise ratio was setting to protein ratio calculation and t-test was chosen as statistical method for group comparison (HC vs. DMSO). Proteins with a log_2_ fold change >+0.58 or < -0.58 and p-value<0.05 were considered up- or downregulated, respectively. Functional characterization of differentially expressed proteins was performed using UniprotKB gene ontology related to cellular component. Moreover, enrichment analysis was performed by Metascape (Zhou et al., 2019).

### 2.9 Cell mito stress test

A375 human melanoma cell line (1 × 10^4^) was seeded in a 96 well Seahorse XF96 tissue culture Microplate (Agilent Technologies, Santa Clara, CA, United States) 24 h before the assay, then oxygen consumption rate (OCR) and extracellular acidification rate (ECAR) were measured using the Seahorse XFe96 Analyzer, as already described (Lofaro et al., 2020). The OCR and ECAR were assessed both at the baseline (before any treatment) and following the sequential administration of specific inhibitors and uncouplers or compounds. Two types of experiments were conducted to evaluate the contribution of HC in mitochondrial function. For the first experiment, 24 h after seeding, 0.1% DMSO or 8.4 μM HC or 1 μM carbonyl cyanide-4-(trifluoromethoxy)phenylhydrazone (FCCP, a powerful uncoupler of mitochondrial oxidative phosphorylation), were added to evaluate the mitochondrial response. FCCP disrupts ATP production by transporting protons across the mitochondrial inner membrane, collapsing the proton gradient, and allowing the measurement of the maximum OCR, which reflects the electron transport chain’s capacity and substrate availability. For the second experiment, 24 h post-seeding, MitoStress test was conducted. First, 1.5 μM oligomycin was added to inhibit proton translocation through the F0/F1 complex, thereby blocking ATP synthesis and lowering OCR. This was followed by the addition of 1 μM FCCP or 8.4 μM HC. Finally, the addition of 0.5 μM rotenone and 0.5 μM antimycin A inhibited mitochondrial respiration by targeting complex I and complex III, respectively, reducing OCR to its minimal value. For both experiments, the results were measured and normalized to protein content. Several bioenergetic parameters were derived from these measurements, as previously described (Lofaro et al., 2020).

### 2.10 JC-1 mitochondrial membrane potential assay

A375 human melanoma cells were seeded in a 4-chamber plate at a density of 1.5 × 10^4^ cells/well in 500 μl culture medium in a 5% CO_2_ incubator overnight at 37 °C. After 24 h, the culture medium was removed, and the cells were cultured for 4 h with: i) DMEM plus 0.1% DMSO; ii) complete DMEM plus 8.4 μM HC; iii) complete DMEM plus 100 μM mitochondrial uncoupler carbonyl cyanide m-chlorophenyl hydrazone (CCCP - ThermoFisher Scientific) which depolarizes the mitochondria. After treatment, to assess mitochondrial membrane potential (ΔΨ_m_), A375 cells were incubated with 4 μM JC-1 (ThermoFisher Scientific) in phenol red free DMEM for 20 min in a 5% CO_2_ incubator at 37 °C, then washed twice with DMEM without phenol red. Finally, 500 μl of phenol red free DMEM were added to each chamber. JC-1 dye was excited with a 488 nm laser wavelength and green and red fluorescence were determined using BP 500–551 and BP 560–651 filters, respectively (Perelman et al., 2012). Imaging was performed with a ×63 Plan-Apo oil immersion objective mounted on a Leica SP8 confocal microscope (Leica, Wetzlar, Germany) equipped with an Okolab incubation chamber.

In parallel, A375 cells were seeded in black 96 well plates at a density of 4 × 10^4^ cells/well in a 5% CO_2_ incubator overnight at 37 °C for 24 h. Prior to the assay, the culture medium was removed, and cells were washed with phenol red free DMEM (Gibco). Then 150 μl of 4 μM JC-1 were added in each well for 20 min at 37 °C. After incubation, cells were washed twice with phenol red free DMEM, followed by the addition of 180 μl of phenol red free DMEM. The baseline fluorescence intensity of the dye (Ex 475 ± 20 nm; Em 590 ± 17.5 nm and 530 ± 15 nm) was measured using GloMax Explorer (Promega). After 15 min, 0.1% DMSO or 8.4 μM HC were added in each well and the fluorescence of the dye was monitored for additional 15 min to evaluate changes in ΔΨ_m_.

### 2.11 Mitochondrial morphological analysis by confocal microscopy

A375 human melanoma cells were seeded in a 4-chamber plate at a density of 1.5 × 10^4^ cells/well in 500 μl culture medium in a 5% CO_2_ incubator overnight at 37 °C. After 24 h, cells were cultured for additional 4 h with: i) DMEM plus 0.1% DMSO; ii) complete DMEM plus 8.4 μM HC; iii) complete DMEM plus 100 μM CCCP. Mitochondrial mass was assessed incubating A357 cells with 100 nM MitoView Green (Molecular Probes) in DMEM without phenol red for 15 min at 37 °C. MitoView Green was excited by white light laser (Ex 490 nm; Em 523 nm). Images were taken with a ×63 Plan-Apo oil immersion objective mounted on a Leica SP8 confocal microscope (Leica) equipped with an Okolab incubation chamber.

### 2.12 ROS detection by CM-H_2_DCFDA and GSH/GSSG redox state testing

A375 human melanoma cells were seeded in a black 96 well plate at a density of 4 × 10^4^ cells/well in a 5% CO_2_ incubator at 37 °C for 24 h. At the time of the assay, the medium was removed, and cells were washed twice with DMEM without phenol red. Then 100 μl of CM-H_2_DCFDA (Molecular Probes, Eugene, OR, United States) were added in each well for 45 min at 37 °C. After incubation, 180 μl of DMEM without phenol red (Gibco) were added in each well and the fluorescence intensity of the dye (Ex 480 ± 30 nm; Em 530 ± 30 nm) was measured at baseline (before different treatments) using GloMax Explorer (Promega). After 15 min, 0.1% DMSO or 8.4 μM HC were added in each well and the fluorescence intensity of the dye was measured for additional 15 min.

Intracellular redox balance was assessed by measuring the ratio between reduced and oxidized glutathione (GSH/GSSG) using the GSH/GSSG-Glo™ luminescence assay (Promega TM344). Cells (1 × 10^4^) were seeded in white 96-well plates and after 24 h were treated with DMSO and HC for 4 h. Following treatment, total and oxidized glutathione levels were measured according to the manufacturer’s instructions. Luminescence signal was measured using a GloMax® microplate luminometer (Promega), and the GSH/GSSG ratio was used as an index of intracellular redox status (Zitka et al., 2012).

### 2.13 Statistical analysis

Data were obtained from three independent experiments, with technical triplicates performed for each condition. Unless otherwise specified, data are represented as box plots. Data distribution was formally tested by Shapiro-Wilk normality test. No data points were excluded from the analyses. All statistical analyses were performed by GraphPad Prism (v. 9.5.0). Comparisons among multiple groups were evaluated by analysis of variance (ANOVA) followed by Tukey’s post-hoc test, whereas differences between two groups was performed by t-test. Linear regression of different conditions was performed comparing slopes and intercepts. A p-value <0.05 was set as statistically significant.

## 3 Results

### 3.1 Hallachrome inhibits cell growth of A375 and SK-MEL-28 human melanoma cell lines

**FIGURE 1:**
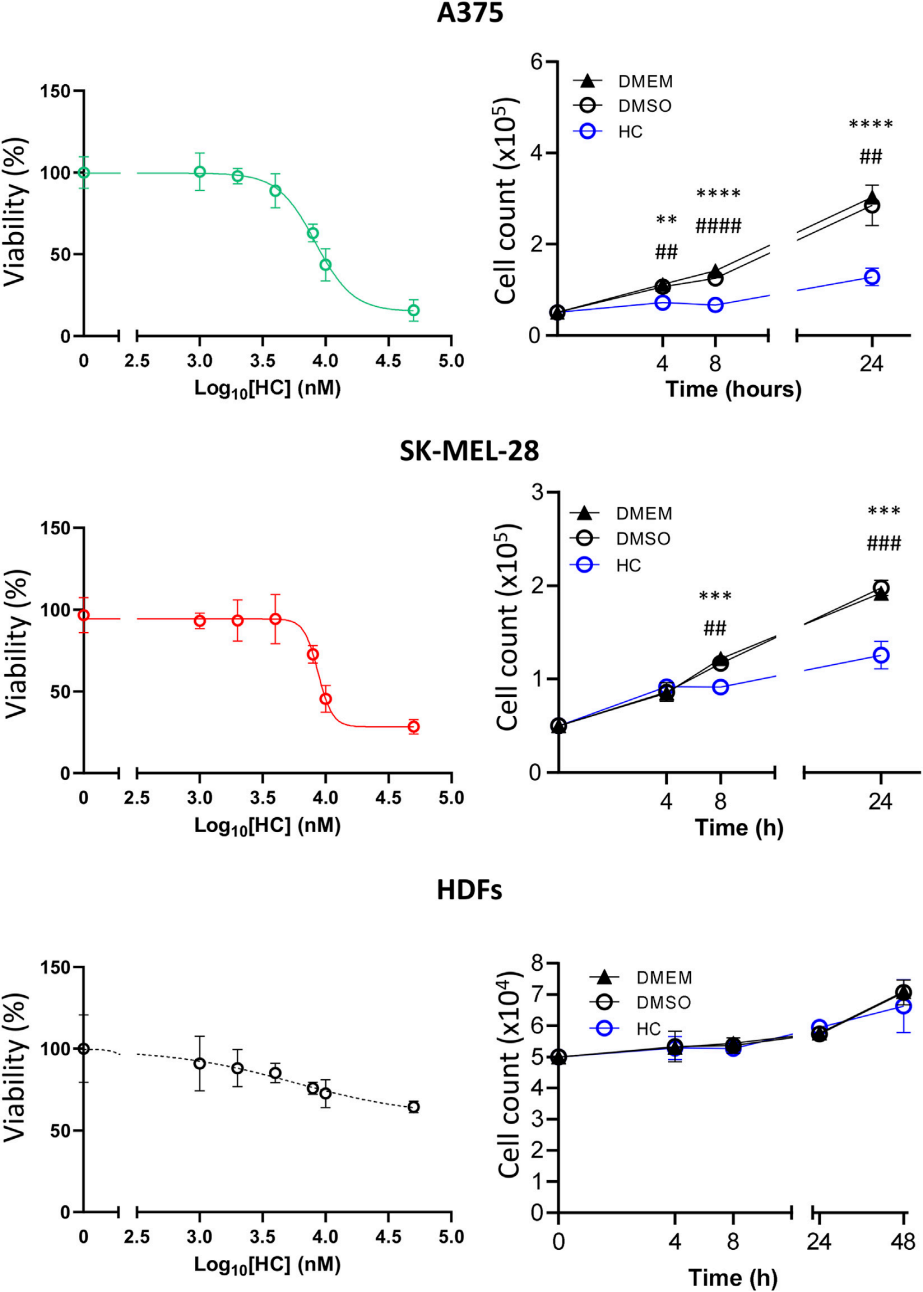
IC_50_ value and cell growth. The dose-response curves show the IC_50_ value of Hallachrome (HC) in A375, SK-MEL-28 melanoma cells and human dermal fibroblasts (HDFs) (left). Viability of A375, SK-MEL-28 melanoma cells and HDFs cultured in DMEM, DMEM plus 0.1% DMSO or in DMEM plus 8.4 µΜ in 0.1% DMSO (right). Values are represented as mean ± standard deviation from three independent experiments for condition, each analyzed in technical triplicate. **p < 0.01; ***p < 0.001; ****p < 0.0001 DMEM vs. HC; ##p < 0.01; ###p < 0.001; ####p < 0.0001 DMSO vs. HC.

To evaluate the effect of HC on cell viability, A375, SK-MEL-28 and primary dermal fibroblasts were treated with different concentrations of the molecule (i.e., 1, 2, 4, 8, 10, 20 and 50 μM) for 24 h. Dose-response analysis revealed a similar IC_50_ value (the drug concentration that inhibited 50% cell viability) in both melanocytic cell lines (A375 = 8.4μM ± 0.2 μM and SK-MEL-28 = 8.7μM ± 0.15 μM) (Figure 1). This value aligns with concentrations tested using other anthraquinones (Zhu et al., 2012). In contrast, it was not possible to estimate the IC50 value for primary human dermal fibroblasts (HDFs), as cell viability remained above 50% even at the highest concentration tested (50 μM) after 24 h of treatment (Figure 1). Light microscopy images showed a concentration-dependent reduction in cell density following HC treatment in A375 and SK-MEL-28 cells, but not in HDFs (Supplementary Figure S1).

Based on these findings and to investigate the early mechanisms by which the compound begins to inhibit cell growth, all cell lines were incubated with 8.4 μM HC, and cell growth was monitored at various intervals over a 24-h period (i.e., 4, 8, and 24 h). Untreated cells and cells treated only with the solvent (i.e., 0.1% DMSO) exhibited similar proliferation rates. In contrast, HC markedly reduced the cell growth over time in A375 and SK-MEL-28 cell lines (Figure 1). Consistent with the dose response analysis, treatment with 8.4 μM HC did not affect the growth of HDFs, even after 48 h of culture. This time point was included because primary HDFs exhibit a longer doubling time (Moulin et al., 2011; Boraldi et al., 2015) compared to melanoma cells (Benga, 2001). In our experimental conditions, HC exerts antiproliferative effects in melanoma cells while showing no detectable impact on the growth of non-malignant fibroblasts, at least at 8.4 μM for 48 h.

A significant reduction in A375 cell growth was already evident after 4 h of treatment. This time point was therefore chosen for further experiments aimed at characterizing the early cellular response induced by HC. Since A375 and SK-MEL-28 cells showed a similar sensitivity to HC, further investigations were performed exclusively in A375 cells. This cell line, derived from a primary amelanotic melanoma, are widely used as an experimental model to investigate melanoma cell proliferation, survival, metabolism, and drug responses (Giard et al., 1973; Shen et al., 2025). In contrast, SK-MEL-28 cells originate from a metastatic melanoma lesion and exhibit additional biological alterations associated with advanced disease progression (Carey et al., 1976; Xing et al., 2012). For this reason, A375 cells were selected to characterize the early cellular effects of HC in a model representative of primary melanoma while minimizing the potential influence of molecular changes associated with metastatic progression.

### 3.2 Hallachrome induces morphological changes in A375 human melanoma cells, inhibit cell migration and apoptosis

**FIGURE 2:**
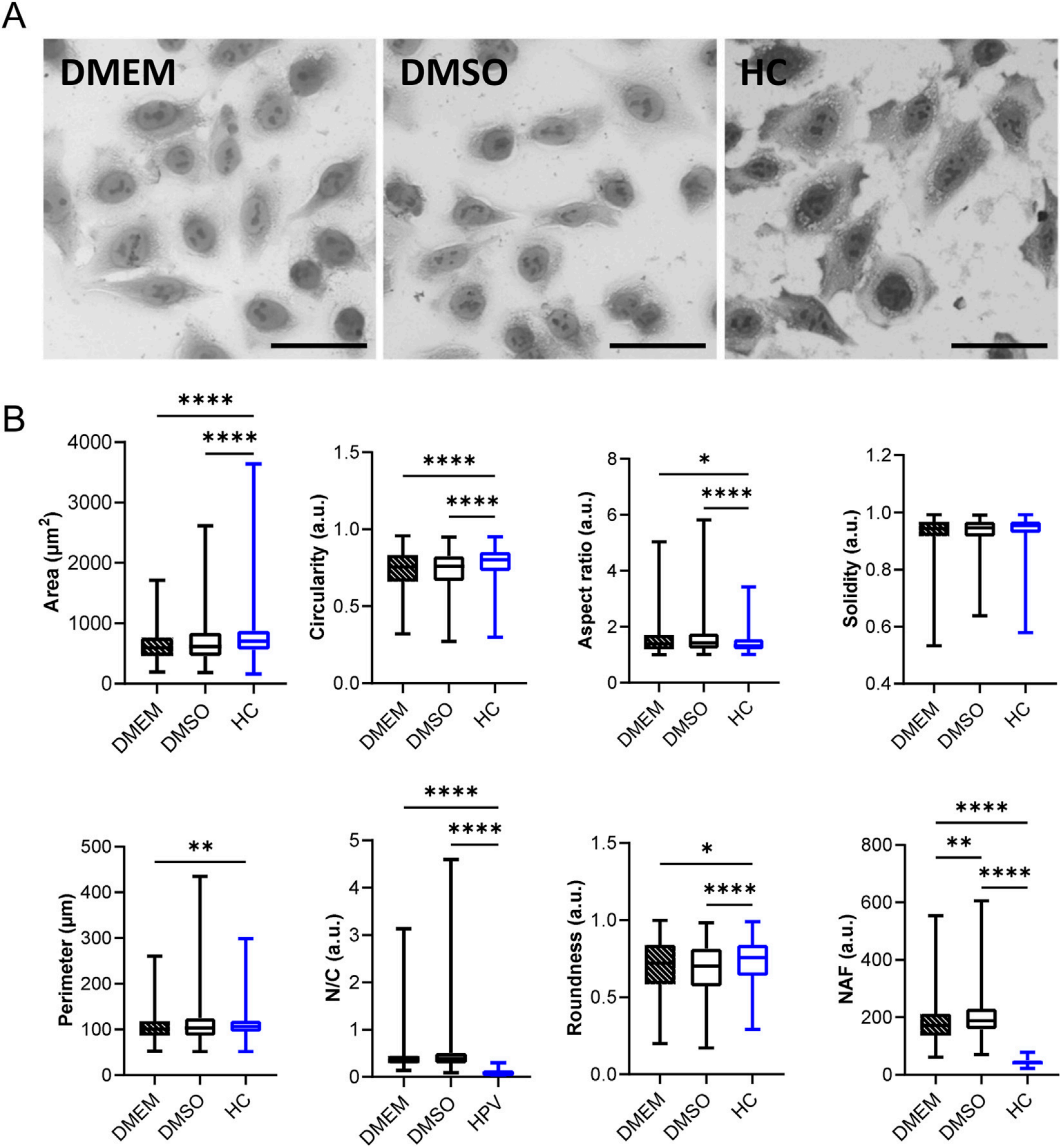
A375 brightfield microscopy images and morphometric analysis. **(A)** Representative image of A375 human melanoma cells cultured in DMEM alone, in DMEM plus DMSO and in DMEM plus HC (Hallachrome) for 4 h. **(B)** Morphometric parameters of A375 human melanoma cells. Data from at least 350 cells for each experimental condition are represented as box plot, which center lines indicating the medians, box limits corresponding to 25th and 75th percentiles. All experiments were performed using three independent experiments for condition, each analyzed in technical triplicate *p < 0.05; **p < 0.01; ****p < 0.0001. Magnification ×20. Scale bar = 50 μm.

To evaluate the cell morphology, cells were fixed with cold methanol and stained with crystal violet. Under light microscopy, the cells cultured with DMEM or with DMSO appeared small with a nucleus occupying most of the cell (Figure 2A). In contrast, A375 cells treated with HC showed numerous cytoplasmic vesicles and appeared larger compared to other experimental conditions (Figure 2A). These observations were confirmed by quantification of cell shape descriptors (Figure 2B). Cells untreated (DMEM) or treated with solvent only (DMSO) did not show significant differences for the various parameters analyzed (Figure 2B). Instead, HC induced a significant increase in the area, and in the perimeter compared to DMEM and DMSO treated cells. Interestingly, the nuclear area factor (NAF), calculated as the product of nuclear area and nuclear roundness, was significantly lower in HC-treated cells than in other experimental conditions.

**FIGURE 3:**
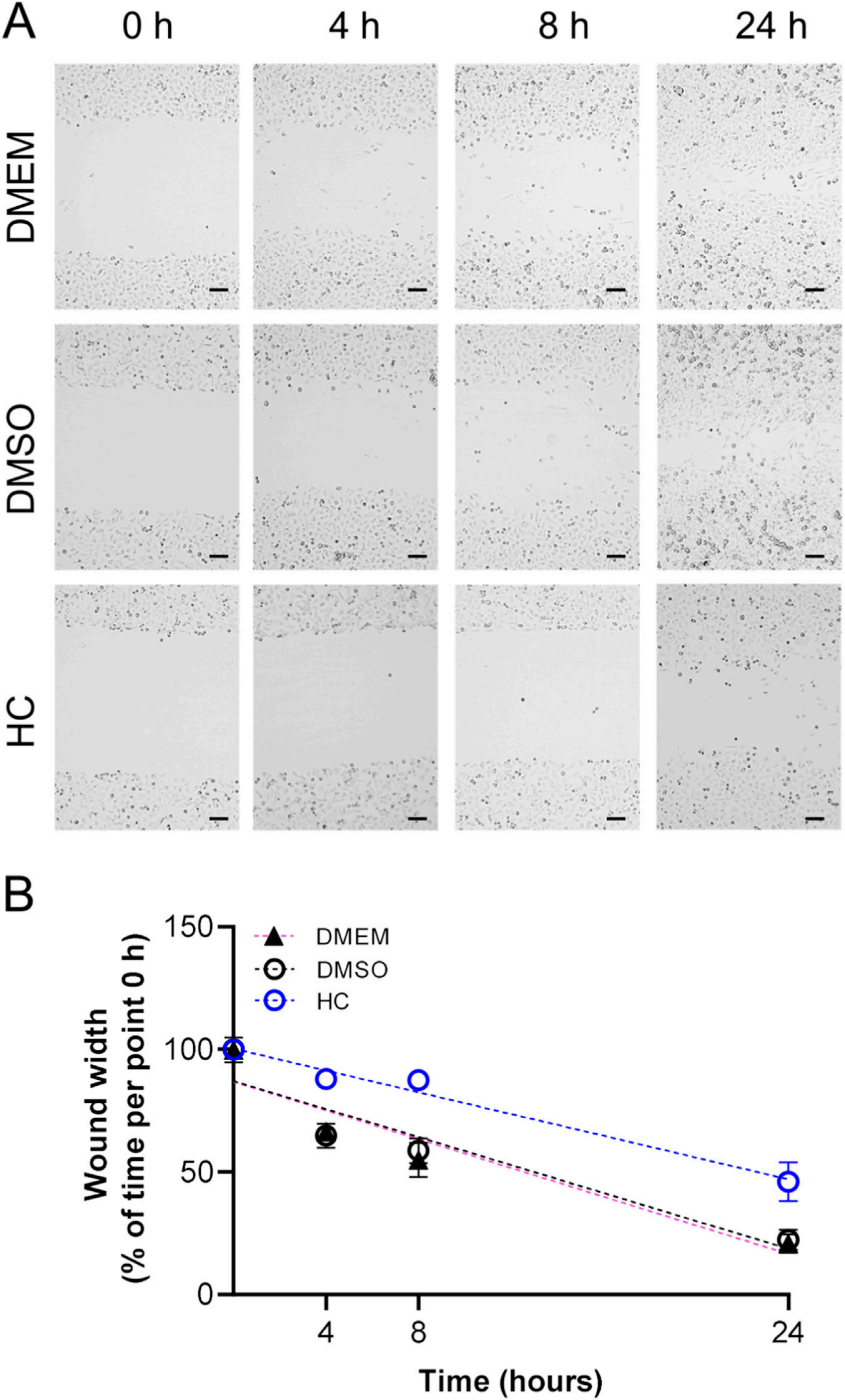
Scratch assay. A375 human melanoma cells incubated with DMEM alone or DMEM plus DMSO or DMEM plus HC (Hallachrome). **(A)** Representative image of the scratch test for 24 h. Images were acquired at three time points after the scratch (0 h). **(B)** Linear regression analysis of *in vitro* wound closure over time, with wound area quantified using ImageJ. Data are represented as mean ± SD and were derived from three independent experiments, each performed in technical triplicate for experimental condition. Magnification ×10. Scale bar = 100 μm.

To assess the effect of HC on melanoma cell mobility, cell migration was evaluated using both scratch wound-healing assay and a Transwell migration test. Time-lapse light microscopy observation of wound closure revealed that A375 cells treated with HC exhibited a reduced migration rate compared to both untreated (DMEM) and DMSO-treated cells. (Figure 3A). Moreover, quantitative analysis of wound width over time revealed a linear relationship for all treatment conditions (y_DMEM_ = −2.997x+88.2; y_DMSO_ = −2.845x+86.9; y_HC_ = −2.124x + 98.3). Notably, the slope of the regression line for HC-treated cells was significantly different from the other two conditions, indicating a reduced migration rate under HC treatment (p-value_DMSOvs.DMEM_ = 0.7157; p-value_HC vs. DMEM_ = 0.0155; p-value_HCvs.DMSO_ = 0.0388) (Figure 3B).

**FIGURE 4:**
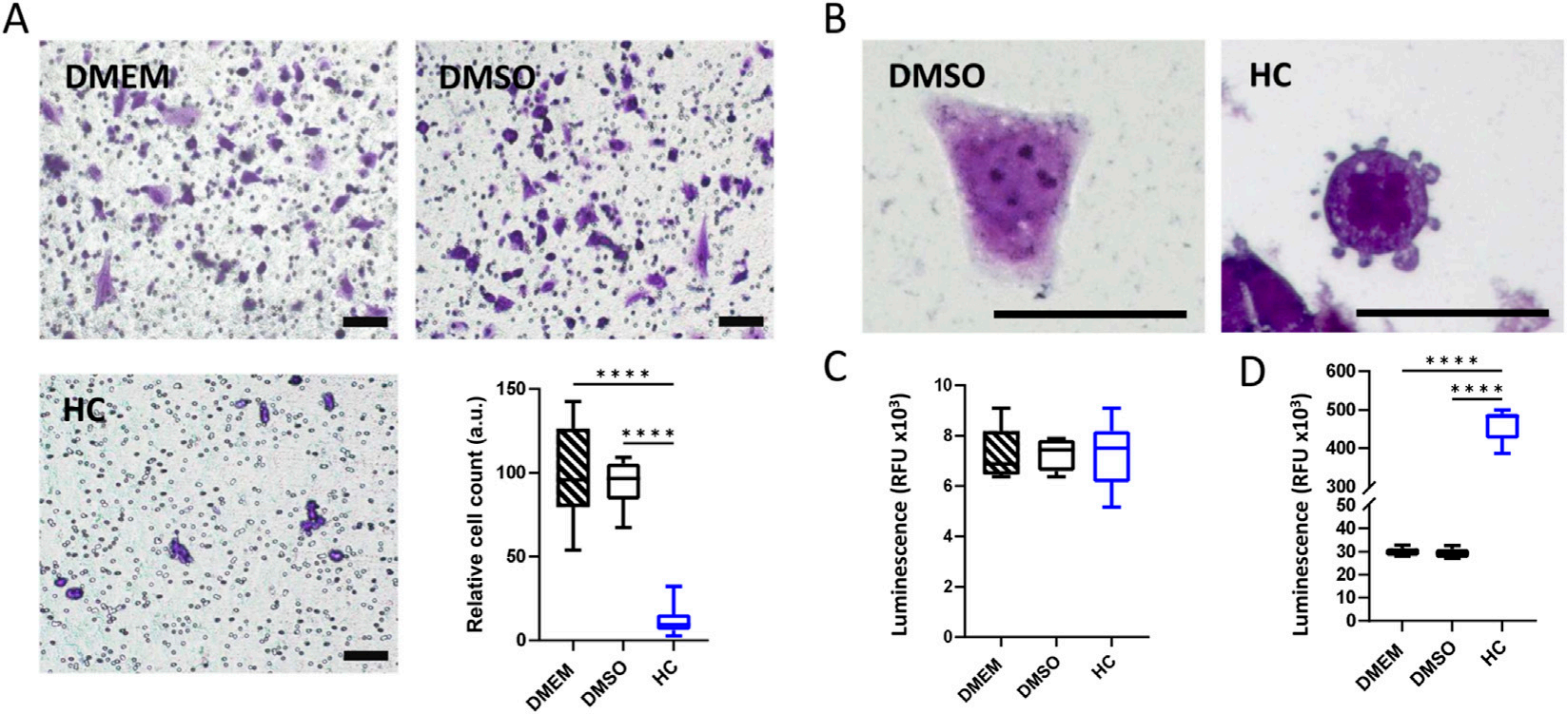
Transwell migration test and apoptosis assay. A375 human melanoma cells incubated with DMEM alone or DMEM plus DMSO or DMEM plus HC (Hallachrome). **(A)** Representative images of crystal violet-stained cells that migrated through Transwell inserts in different experimental conditions. Quantification of migrated cells is shown in the graph. Cells cultured in DMEM were set to 100%. **(B)** Representative image of A375 cells stained with crystal violet after 24 h of DMSO or HC treatment. HC induced apoptosis. **(C,D)** Apoptosis quantification using GloMax® microplate reader after 4 and 24 h of treatment, respectively. RFU = Relative Fluorescence Units. Data from three independent experiments, each performed in technical triplicate for experimental condition, are represented as box plot, which center lines indicating the medians, box limits corresponding to 25th and 75th percentiles. ****p < 0.0001. Magnification ×10 (A) and 25X (B). Scale bar = 50 μm.

The inhibitory effect of HC on cell mobility was further confirmed by Transwell migration test. Consistent with the wound-healing results, HC treatment significantly reduced the number of migrating cells in comparison with cells treated with both DMEM and DMSO (Figure 4A), supporting the conclusion that HC impairs the migration potential of A375 melanoma cells.

Given the marked effects of HC on cell proliferation and migration, we next assessed whether HC treatment triggered apoptotic cell death. No significant differences were observed after 4 h of treatment; however, after 24 h, HC-treated cells exhibited a significant increase in apoptosis compared with both the DMEM and DMSO conditions (Figures 4B–D).

### 3.3 Proteomic analysis of hallachrome treated cells

**FIGURE 5:**
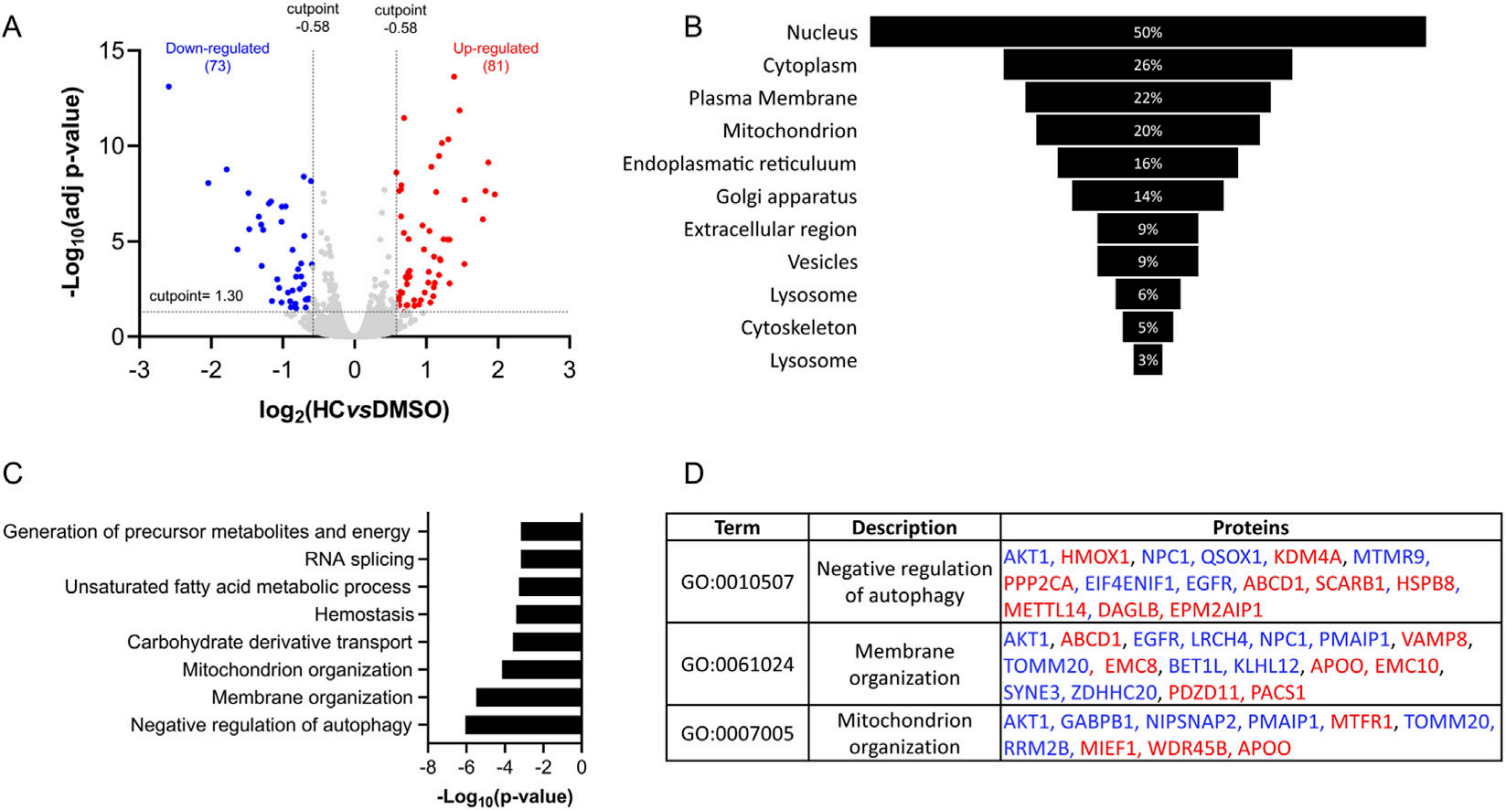
Proteomic analysis. **(A)** Volcano plot, the X-axis represents the log_2_ values of the fold change (FC) observed for each protein, and the Y-axis depicts the -log_10_ of the p-values of the significance t-tests. Proteins with log_2_ FC (HC vs. DMSO) > 0.58 (red) or < −0.58 (blue) and with a p-value<0.05 are considered differently expressed proteins (DEPs) and colored in red and in blue for up- and downregulated proteins, respectively. Unchanged proteins are represented in gray. All experiments were performed using three independent experiments, each analyzed in technical triplicate for condition. **(B)** Localization of DEPs through different cellular compartments. **(C)** Histogram of enriched GO terms of DEPs. **(D)** Top three enriched GO terms obtained through Metascape platform analysis.

Since A375 melanoma cells exhibited morphological modifications and an altered migration capability after 4 h of treatment, we performed a label free proteomic analysis by mass spectrometry on DMSO- and HC-treated A375 melanoma cells. Cells cultured in DMEM alone were excluded from the following analysis, because, in the experiments described above, they showed no differences compared to DMSO-treated cells. We identified 5,550 proteins with high confidence across at least one condition in six technical replicates (Supplementary Table S1). Label-free quantification, based on precursor ion intensity, was performed to compare the relative abundance of proteins between two experimental conditions. This analysis revealed 154 differentially expressed proteins (DEPs) (Supplementary Table S2), of which 81 and 73 were up- and downregulated, respectively, in HC-treated cells (Figure 5A). To determine the subcellular localization of DEPs, we used UniProtKB database. While few proteins were exclusively localized in a single compartment (e.g., RBBP9 and PLD3B), most DEPs were distributed across different compartments (Figure 5B).

Furthermore, enrichment analysis was conducted by Metascape platform: DEPs were significantly enriched in negative regulation of autophagy (GO:0010507); membrane organization (GO:0061024); mitochondrial organization (GO:0007005) (Figures 5C,D).

Specifically, HC induced a significant change in 15 proteins (i.e., 9 and 6 up- and down-regulated, respectively) involved in autophagy, including the down-regulation of NPC1 (Nieman-Pick type C) and QSOX1 (quiescin sulfhydryl oxidase 1) and the up-regulation of HMOX1 (heme oxygenase 1) and KDM4A (lysine demethylase 4A). Regarding membrane organization, 17 proteins were affected, including the down-regulation of EGFR (epidermal growth factor receptor) and AKT1 (protein kinase B) and the up-regulation of APOO (apolipoprotein O) and PACS1 (phosphofurin acidic cluster sorting protein-1) (Figure 5D). Enrichment analysis using Metascape also identified 10 DEPs involved in mitochondrial organization (i.e., 4 and 6 were up- and downregulated, respectively, after HC treatment) (Figure 5D) with a downregulation, for example, of AKT1, TOMM20 (translocase of the outer mitochondrial membrane 20), and RRM2B (Ribonucleotide Reductase Regulatory TP53 Inducible Subunit M2B), and an upregulation of MTFR1 (mitochondrial fission regulator 1) and MIEF1 (mitochondrial elongation factor 1).

### 3.4 Hallachrome affects mitochondrial function

**FIGURE 6:**
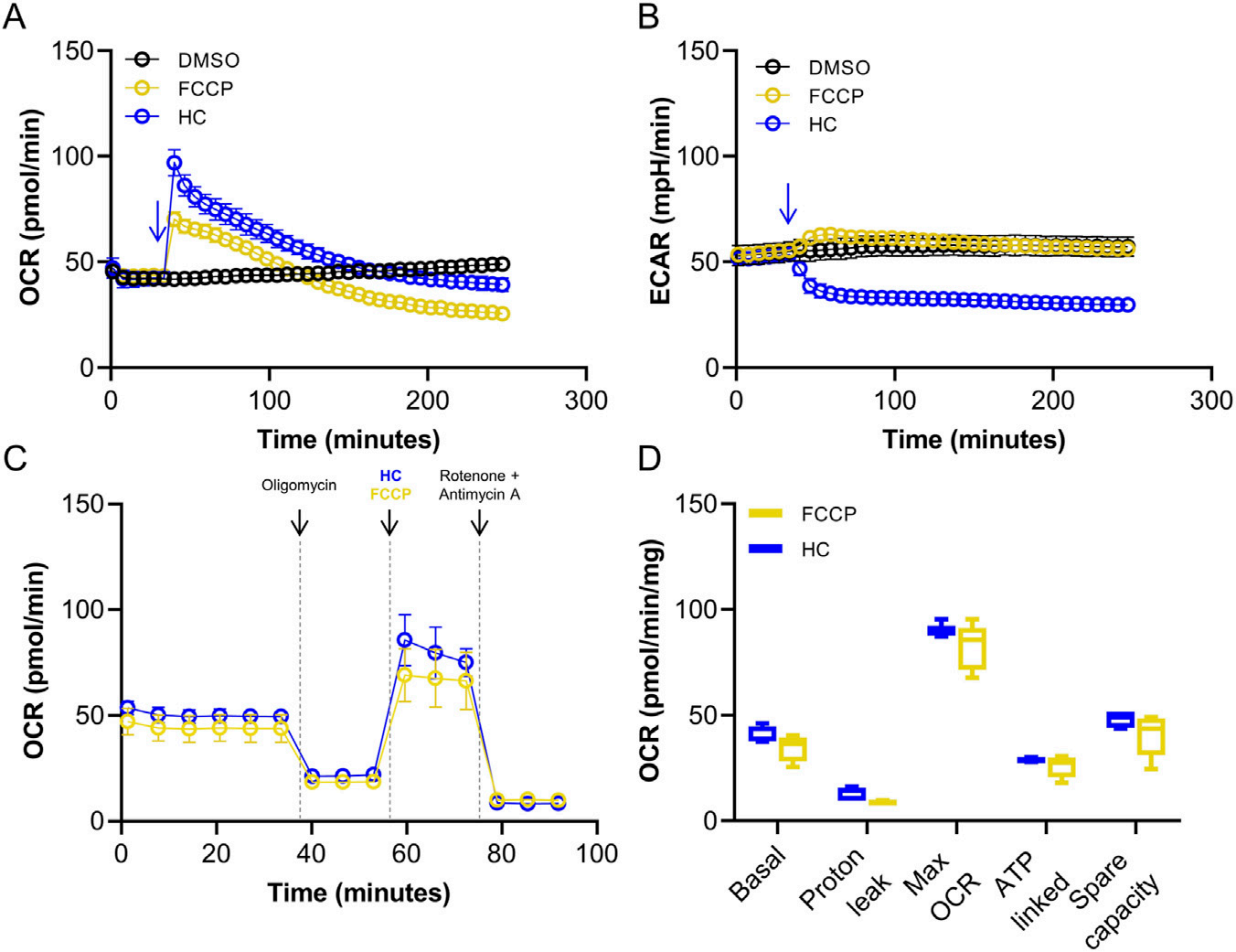
Real-time measures of oxygen consumption rate (OCR). **(A,B)** Quantification of the basal OCR and extracellular acidification rate (ECAR) in A375 human melanoma cells treated with DMSO or Hallachrome (HC) or carbonyl cyanide-4-(trifluoromethoxy)phenylhydrazone (FCCP). Blue arrows indicate DMSO or HC or FCCP injection. **(C)** Results from the MitoStress test of A375 human melanoma cells. To evaluate mitochondrial function, cells were subsequently injected with oligomycin, FCCP or HC, and then with rotenone/antimycin A. Black arrows indicate the different compounds injection. **(D)** Quantification of the basal OCR, proton leak, maximal OCR, ATP-linked OCR and spare capacity in HC- and FCCP-treated A375. All data derived from three independent experiments, each analyzed in technical triplicate for every experimental condition, are represented as box plot, which center lines indicating the medians, box limits corresponding to 25th and 75th percentiles.

Seahorse technology provides real-time information about the OCR, which reflects the ability of mitochondria to produce ATP. Interestingly, the OCR dramatically rises immediately after HC injection, followed by a progressive decline. Comparable values were observed with FCCP administration (Figure 6A), supporting that HC may act as a mitochondrial uncoupler. Moreover, we detected a reduction in extracellular acidification rate (ECAR) (Figure 6B), suggesting a diminished glycolytic activity.

To further investigate HC as a potential mitochondrial uncoupling agent, we conducted a MitoStress assay, using sequential injections of specific compounds to assess mitochondrial function under varying metabolic conditions (see Methods). Figure 6C shows the OCR profile after FCCP injection. A comparable response was obtained when HC was injected instead of FCCP. Respiratory parameters (e.g., basal respiration, maximal capacity) did not differ significantly between the two experimental conditions (Figure 6D), reinforcing the hypothesis that HC acts as a mitochondrial uncoupler.

**FIGURE 7:**
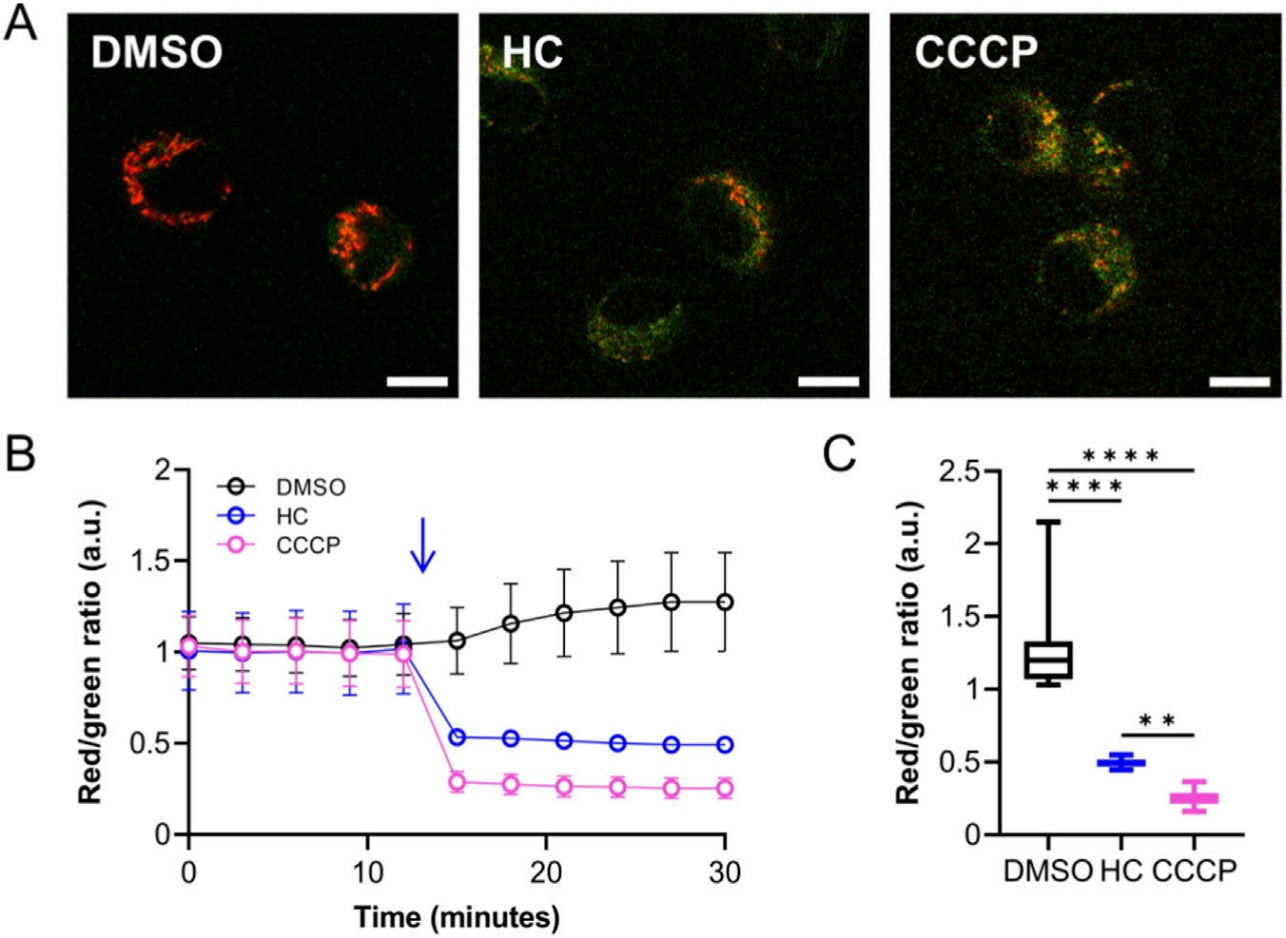
Mitochondrial membrane potential. **(A)** A375 human melanoma cells cultured with DMSO, or HC (Hallachrome) or CCCP (carbonyl cyanide m-chlorophenyl hydrazone), stained with JC-1 and observed by confocal microscopy in a live-imaging mode. **(B)** Real-time measurement of red/green fluorescence ratio of JC-1 before and subsequently the administration of DMSO or HC. Blue arrows indicate DMSO or HC injection. **(C)** Histogram showing the ratio of red to green fluorescence intensity of JC-1 staining measured 15 min after DMSO or HC injection. All data derived from three independent experiments, each performed in technical triplicate for experimental condition, are represented as box plot, which center lines indicating the medians, box limits corresponding to 25th and 75th percentiles. **p < 0.01, ****p < 0.0001. Magnification 63X. Scale bar = 10 μm.

To investigate the effect of HC on mitochondrial membrane potential (ΔΨm), we used JC-1, a lipophilic cationic dye that accumulates in mitochondria in a potential-dependent manner. In polarized mitochondria, JC-1 forms red fluorescent J-aggregates, while depolarized mitochondria retain green fluorescence monomers (Cossarizza and Salvioli, 1998). A375 cultured in DMSO displayed predominantly red fluorescence indicative of polarized mitochondria (Figure 7A). Instead, HC induced a reduction of red fluorescence compared to the DMSO condition similar to CCCP, a known mitochondrial membrane disruptor which causes a complete mitochondria depolarization (Figure 7A). To confirm this observation, we quantitatively assessed mitochondrial membrane potential by measuring the red/green fluorescence ratio of JC-1 following DMSO or HC injection. The results demonstrated a significant reduction in this ratio upon HC and CCCP administration, indicating mitochondrial depolarization (Figures 7B,C).

**FIGURE 8:**
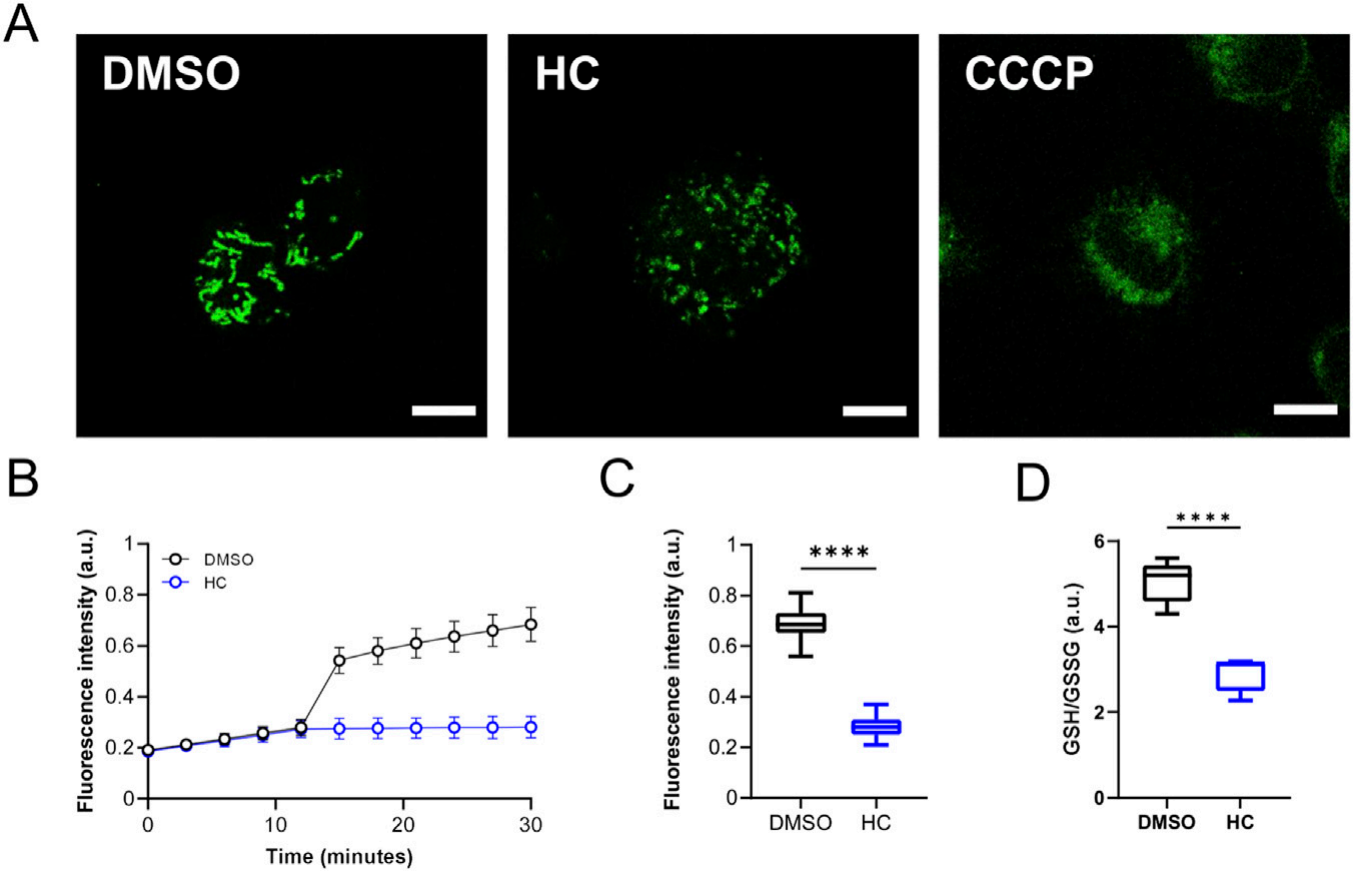
Mitochondrial morphology, ROS levels detection and GSH/GSSG ratio. **(A)** A375 human melanoma cells cultured with DMSO, or HC (Hallachrome) or CCCP (carbonyl cyanide m-chlorophenyl hydrazone), stained with MitoView and observed by confocal microscopy in a live-imaging mode. **(B)** Real-time measurement of fluorescence intensity of A375 stained with CM-H_2_DCFDA before and after the administration of DMSO or HC. Blue arrows indicate DMSO or HC injection. **(C)** Histogram showing the fluorescence intensity of CM-H_2_DCFDA staining measured 15 min after DMSO or HC injection. **(D)** GSH/GSSG ratio after 4 h of DMSO or HC treated cells. All data derived from three independent experiments; each performed in technical triplicate for each experimental condition. Histograms are shown as box plot, where the center line indicates the median and box limits correspond to 25th and 75th percentiles. ****p < 0.0001. Magnification 63X. Scale bar = 10 μm.

Moreover, our data indicate that HC impairs mitochondrial membrane potential in A375 melanoma cells, suggesting a loss of electrochemical gradient and increased membrane permeability. Given the tight relationship between mitochondrial function and morphology (Picard et al., 2013), we investigated the mitochondrial morphology using Mitoview Green, a dye that selectively stains mitochondria in live cells in a voltage-independent manner. In particular, mitochondria in DMSO-treated cells displayed an elongated morphology, while HC- and CCCP-treated cells showed rounder and smaller mitochondria, indicative of isolated and fragmented organelles (Figure 8A).

To assess whether HC affected intracellular ROS level, we used CM-H_2_DCFDA, a fluorescent dye sensitive to oxidative stress. Interestingly, HC blocked the progressive increment of H_2_O_2_ observed over time in DMSO condition (Figures 8B,C).

To further investigate the effects of HC on cellular redox homeostasis, the GSH/GSSG ratio, a hallmark of a more oxidized intracellular redox state (Zitka et al., 2012), was measured. A significant decreased of the GSH/GSSG ratio was observed following HC treatment (Figure 8D), suggesting an impairment of the cellular reducing capacity and a shift toward a more oxidizing redox state.

## 4 Discussion

Treatment with HC leads to significant changes in melanoma cell viability, morphology and migration, accompanied by early molecular and functional responses. In particular, HC induced a significant inhibition of cell growth with an increase of cell size and cytoplasmic vesicle and pronounced nuclear remodeling, commonly observed in cells undergoing stress-associated growth arrest (Lloyd, 2013; Matos and Jordan, 2015). This phenotype is consistent with the concept that smaller cells are primarily engaged in proliferation, whereas larger cells often shift to alternative metabolic activities, diverting energy from replication to other cellular processes (Vander Heiden et al., 2009). Among the parameters commonly altered in cancerous cells is N/C ratio, also known as the karyoplasmic ratio. Alteration in the N/C ratio are associated with enhanced migration and invasive capacities (Rizzotto et al., 2020). HC significantly reduced the N/C ratio and decreased the migration capacity of A375 cells. Importantly, these early phenotypic alterations preceded the induction of apoptosis, which became significantly evident after 24 h of HC treatment. These data suggests that HC initially trigger a stress-associated growth arrest accompanied by morphological remodeling and reduced motility, followed by the activation of apoptotic pathways upon prolonged exposure.

These results are in agreement with previous studies showing reduced cell growth and mobility after treatment with other anthraquinones (Abu et al., 2013; Li et al., 2023; Xue et al., 2023), further supporting the role of this class of molecules in modulating cancer cell behavior.

In addition to these effects, HC altered the expression of several proteins associated with autophagy-related processes. Autophagy is a key biological process where cells degrade damaged DNA, proteins or organelles to maintain cellular homeostasis or respond to stress or starvation (Liu et al., 2023). Its role in cancer is debated since both tumor-inhibiting and tumor-promoting functions of autophagy have been reported (Mathew and White, 2007; Galluzzi et al., 2015; Amaravadi et al., 2016). Moreover, autophagy interacts with the cell death process and can influence the responsiveness of cancer cell to therapeutic treatments (Mulcahy Levy and Thorburn, 2020). In our experimental condition, HC treatment determined a downregulation of NPC1, a lysosomal cholesterol transporter that facilitates the efflux of cholesterol from the lysosome to the cytoplasm. It has been demonstrated that the loss of NPC1 impairs the proper function of the autophagy process (Sarkar et al., 2013). At the contrary, increased NPC1 expression has been associated with enhanced migration and proliferation in hepatocellular carcinoma by activating autophagy (Xu et al., 2024). HC also reduced the expression of QSOX1, an enzyme catalyzing the disulfide bonds formation. This enzyme is involved in protein folding and stability (Pernodet et al., 2012) and several studies have already demonstrated that an over-expression of QSOX1 in different cancer type is also associated with an aggressive disease. On the contrary, loss of QSOX1 significantly impair tumor cell proliferation and invasiveness (Katchman et al., 2013). Consistent with these observations, HC treated cells displayed reduced proliferative and migratory capacities.

In addition, HC treatment significantly upregulated the expression of HMOX1 and KDM4A, both of which are established mediators of cellular stress responses and autophagy (Banerjee et al., 2012; Bernard et al., 2015; Busserolles et al., 2006). Notably, the induction of HMOX1 likely serves as a compensatory antioxidant response to mitigate the mitochondrial and metabolic stress triggered by HC. Although autophagy activity and flux were not directly assessed in the present study, proteomic profiling identified a subset of DEPs previously linked to autophagy-related pathways, suggesting the engagement of autophagy-associated molecular processes.

Beyond autophagy-related proteins, HC altered the expression of proteins involved in membrane organization and signaling transduction. In particular, EGFR, a tyrosine kinase receptor of the ErbB family, activates multiple downstream signaling pathways (Oda et al., 2005) such as RAS/MAPK(ERK) and PI3K/AKT/mTOR pathways that collectively promote cell proliferation and survival while inhibit apoptosis. These pathways are often dysregulated in many cancers (Roskoski, 2014), underscoring the relevance of their modulation following HC treatment. Moreover, AKT1, a serine/threonine kinase downregulated by HC, is crucial for cellular processes, including cell cycle progression, genome stability, and protein synthesis (Szymonowicz et al., 2018). Particularly, AKT1 phosphorylates various proteins localized in the nucleus, cytosol and plasma membrane thus promoting cell proliferation, survival, migration and invasion (Liu et al., 2009; Hers et al., 2011). Acting as a central node within the EFGR/PI3K/AKT/mTOR signaling axis, AKT1 dysregulation has been linked to different pathological conditions such as cancer, neurological diseases and diabetes (Hers et al., 2011) and it is recognized as an oncogene in melanoma (Wei et al., 2019).

In addition to canonical signaling molecules, HC treatment led to the upregulation of APOO and PACS1. Recent studies have shown that some APOs are involved in key cancer pathways (e.g., PI3K/AKT) and play roles in regulating inflammation, proliferation and apoptosis (Wang C. et al., 2021; He et al., 2022). Currently, there are no information regarding APOO in cancer, highlighting the need for further studies to elucidate its function, particularly in melanoma. PACS1, a protein involved in membrane trafficking regulation and replication (Mani et al., 2020), has recently emerged as a pivotal regulator of the BAX/BAK-dependent mitochondrial apoptosis pathway, controlling key steps in cell fate decisions (Brasacchio et al., 2017). Interestingly, in our experiments, HC treatment induced a cellular stress response followed by a significant increase in apoptosis after 24 h of treatments. Taken together, these findings suggest that HC may impact not only on signaling pathways but also membrane associated mechanisms governing survival and apoptosis.

Interestingly, HC also altered the expression of proteins involved in mitochondrial organization and function. This finding was not unexpected, as several studies have demonstrated that mitochondrial dysfunction is linked to cancer development and progression and activation of programmed cell death (Srinivasan et al., 2017; Nguyen et al., 2023).

As previously reported, AKT1 plays a key role in several cellular processes, including the regulation of mitochondrial dynamics by influencing the expression of proteins related to mitochondrial fusion and fission (Xie et al., 2022). Previous studies have demonstrated that the AKT pathway activation can inhibit mitochondrial fission, promoting cancer cell migration, and proliferation (Xie et al., 2022). Giving the central role of mitochondria in ATP production and ROS generation, the reduction of AKT1 expression observed after HC treatment may significantly affect the cellular energy balance and redox status. In this contest, AKT1 downregulation can lead to impaired mitochondrial dynamics, contributing to mitochondrial damage with a reduction of cell growth, as revealed both in our study and in other experimental conditions (Xie et al., 2022). Consistently, the alteration of TOMM20 expression observed in HC-treated cells may further support mitochondrial involvement. Indeed, TOMM20 over-expression promotes cell proliferation and migration through enhanced ATP synthesis, whereas its downregulation impairs mitochondrial dysfunction (Park et al., 2019). Additionally, HC treatment induced the upregulation of MTFR1 and MIEF1, proteins directly involved in mitochondrial fusion/fission, as well as in cell death, cancer cell proliferation, and metabolism (Wang et al., 2015; Li et al., 2021). In particular, mitochondrial fission plays a crucial role in preserving mitochondrial integrity, since it can separate damaged or depolarized mitochondria from healthy organelles. Therefore, dysregulation of this process may comprise mitochondrial integrity and cellular viability. In parallel, HC induced the downregulation of RRM2B, a protein involved in mitochondrial DNA synthesis (Pontarin et al., 2012). Reduced levels of this protein may impair mitochondrial genome stability, potentially contributing to mitochondrial dysfunction.

Since our proteomic study suggested mitochondrial alterations following HC treatment, and considering the recent hypothesis of Simeone (Simeone, 2025) that HC might interact with key cellular antioxidant systems, mitochondrial function was further investigated through oxygen consumption rate (OCR), extracellular acidification rate (ECAR), mitochondrial membrane potential, mitochondrial network morphology, ROS production and GSH/GSSG ratio. Mitochondria functional assays demonstrated that HC modulated OCR in manner comparable to the mitochondrial uncoupler FCCP, supporting the hypothesis that HC acts as a functional mitochondrial uncoupler, similarly to other anthraquinones (Simeone, 2025). Although cancer cells typically show a metabolic shift toward increased glycolysis and reduced oxidative phosphorylation, a metabolic adaptation known as the “Warburg effect”, they still depend on mitochondrial ATP production for survival (Weinberg and Chandel, 2015; Gill et al., 2016). Consequently, HC-induced mitochondrial impairment could contribute to metabolic stress potentially promoting cell death (Wang et al., 2024). In agreement with this hypothesis, Sugiyama and collaborators demonstrated that a plant derived anthraquinone significantly reduced ATP levels in melanoma cells, slowing down cellular growth (Sugiyama et al., 2019). Consistently with a mechanism involving mitochondrial uncoupling, HC-treated cells showed a Δψm drop comparable to that observed in CCCP-treated cells. Given the tight relationship between mitochondrial function and morphology (Fenton et al., 2021), loss of membrane potential is known to promote mitochondrial fragmentation (Petit et al., 1995), that has been observed in HC-treated cells. Mitochondrial uncoupling has also been associated with altered ROS production (Pardo-Andreu et al., 2011; Kenwood et al., 2014). In our experimental condition, HC treatment reduced H_2_O_2_ levels. This result is consistent with previous studies demonstrating that uncoupling agents, in absence of additional respiratory chain inhibitors, can reduce mitochondrial ROS generation (Longo et al., 1999; Brookes, 2005). Mechanistically, dissipation of Δψm reduced electron leakage from respiratory chain, thereby decreasing the formation of superoxide anion and its conversion into H_2_O_2_. Despite the reduction in H_2_O_2_, HC significantly decreased the GSH/GSSG ratio, indicating impairment of intracellular redox homeostasis. One possible explanation is related to the profound bioenergetic alterations caused by mitochondrial uncoupling. Regeneration of reduced glutathione depends on NADPH availability, and mitochondrial NADPH production is partially sustained by nicotinamide nucleotide transhydrogenase (NNT) (Ronchi et al., 2016), which activity is driven by proton motive force. Previous studies have demonstrated that dissipation of Δψm can impair NNT-dependent NADPH generation and compromise mitochondrial antioxidant defenses (Regan et al., 2022). This mechanism, although NADPH levels were not directly measured in the present study, may contribute to the decrease in the GSH/GSSG ratio observed following HC treatment.

Data from the present study are based on *in vitro* evidence in intact cells. Therefore, further studies on isolated mitochondria and *in vivo* models are required to provide additional evidence on the impact of proton-efflux and on the pharmacokinetics or the systemic safety of HC. Moreover, regarding the signaling pathways modulated by HC, a better understanding of their activation and regulation can be offered by phosphoproteomic analysis that, however, requires a strategy different from the one used in the present study (Needham et al., 2019).

Despite these limitations, this study presents important strengths. Indeed, by combining cellular, proteomic and mitochondrial functional analyses, we obtained complementary lines of evidence that consistently point to mitochondria as a major target of HC activity. The concordance between the proteomic profile and the alterations observed in oxygen consumption, mitochondrial membrane potential, mitochondrial morphology, cellular redox balance, proliferation, migration, and apoptosis provides a coherent framework for unravelling the biological effects of this compound. The growing interest in mitochondria-target therapeutic approaches reflects the central role of these organelles in cancer development and progression. Therefore, several mitochondria-focused compounds are being evaluated in both preclinical and clinical investigation. In particular, mitochondrial uncoupling has emerged as a promising strategy due to its ability to disrupt metabolic flexibility, induce energetic stress, and alter cellular redox regulation in cancer cells. Although the use of first-generation uncouplers (e.g., dinitrophenol, DNP) was limited by significant toxicity, subsequent experimental studies and early clinical observations have highlighted the potential benefits of controlled mitochondrial uncoupling in both metabolic diseases and cancer (Goedeke and Shulman, 2021; Shrestha et al., 2021) suggesting that inducing a mild degree of uncoupling may enhance antitumor activity, particularly when combined with conventional chemotherapeutic agents.

Within this framework, the mitochondrial dysfunction induced by HC warrants further investigation to clarify its potential as a mitochondria-targeting anticancer agent.

## Data Availability

The data presented in the study are deposited in the PRIDE repository, accession number PXD081058.
