# Supplementary material for: Hallachrome, a marine anthraquinone, impairs cell proliferation by acting as a functional mitochondrial uncoupler in A375 melanoma cells
Source: Front Pharmacol. 2026 Jul 27;17:1844144. doi: 10.3389/fphar.2026.1844144 (PMC13474988; doi:10.3389/fphar.2026.1844144)
Supplement: Supplementary file 1 [file Image1.pdf]

Supplementary Figure

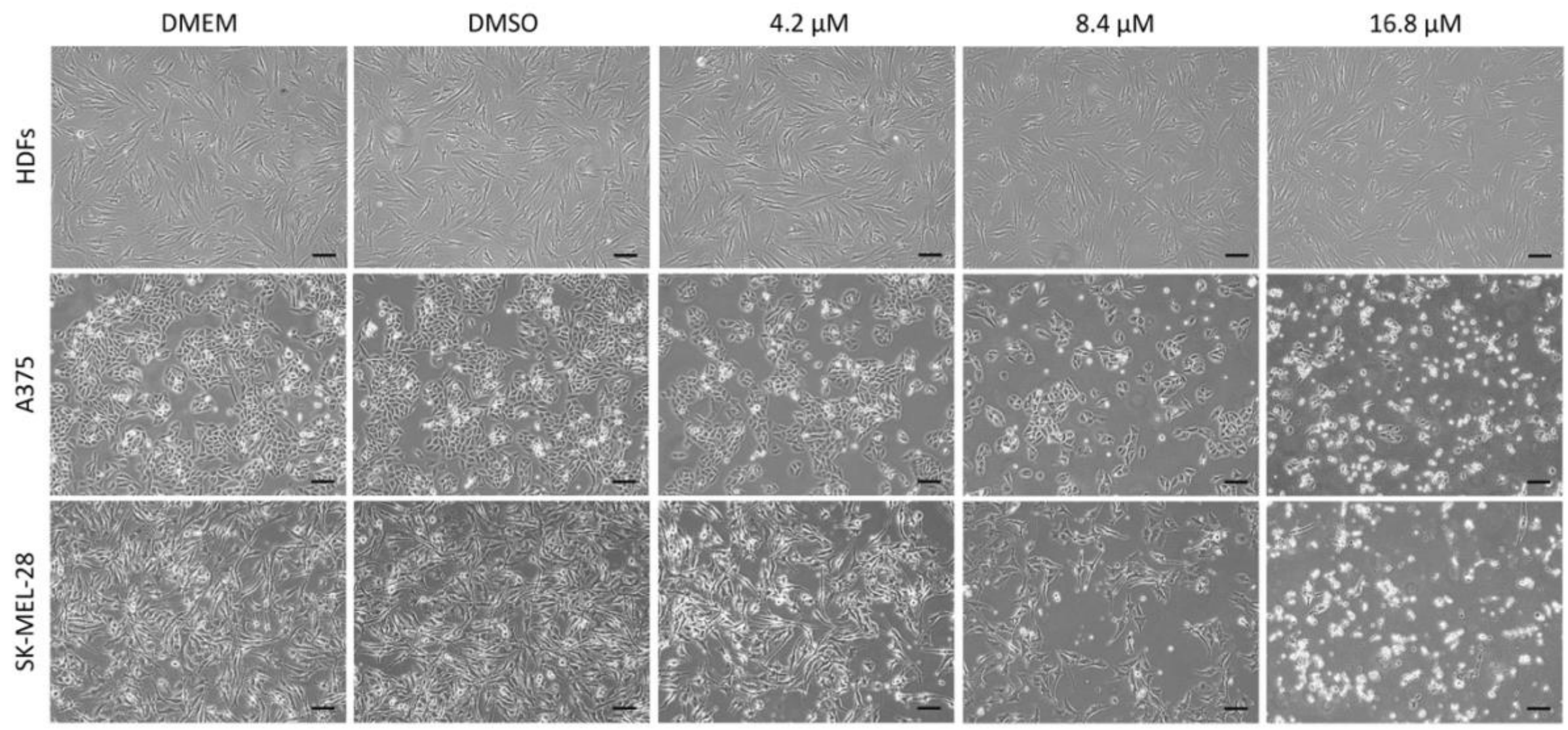

Supplementary Figure 1. Effects of Hallachrome on human dermal fibroblasts and melanoma cells. Representative bright-field microscopy images of human dermal fibroblasts (HDFs), A375 and SK-MEL 28 after 24 hours of culture in DMEM, 0.1% DMSO or in Hallachrome (HC) at concentration corresponding to ½ IC50, IC50 e 2x IC50. The IC50 value established in A375 cells (*i.e.,* 8.4 µM) was applied to all cell lines. No differences were observed between DMSO-treated cells and the DMEM condition in any of the cell lines. HC treatment caused a concentration-dependent reduction in cell density in A375 and SK-MEL-28 cells, whereas no evident changes were detected in HDFs. Magnification 10X. Scale bar: 100 µm
